# Epidemic Cholera

**Published:** 1854-01

**Authors:** J. Haines


### *Epidemic Cholera.*

BY J. HAINES, M. D.

In the year 1817, the Cholera broke out with great violence in India, entered Persia, passed thence to Russia, Poland, Germany, and arrived in England in 1831, being about fourteen years from its eruption in India, until it reached the latter country. Continuing its course westward, it reached this country the year following, taking also a more southernly direction, and visiting France and Spain the same season.

Very little, if anything, was known concerning this disease in this country or Europe, prior to the above date; yet in India they appear to have been familiar with it from a much earlier period, it having visited that country again and again. It will be observed that its general course was from east to west; and that every country, climate and nation was alike subject to its influence. Neither mountains, ocean, nor adverse winds appear to have changed its direction, nor materially checked its progress. Heat and cold, moisture and dryness, were alike subservient to its spread and poisonous effect upon mankind.

It will be recollected that in 1831–2, it was preceded by epidemic influenza, both in Europe and in this country, which to a greater or less extent afflicted almost the entire population in each country. Its march was rapid; nor was it the occasion of serious or permanent injury to any one, except perhaps an occasional pulmonary subject, whom it may have hastened to the tomb. Like the wind before a storm, it appeared to give warning of the approach of something more terrible. For a brief period, like mildew, it poisoned the atmosphere.

Physicians of that day as well as the present, are accustomed to speak of these epidemics as in some measure connected. In many respects certainly they were very dissimilar; but that there was some kindred *miasm* in these maladies, few will doubt. Unlike the influenza, the *Cholera* attacked but a small portion of the whole population, but of the number stricken, the mortality was very great. Twice since then has cholera visited Europe and the United States, yet no influenza accompanied or preceded it.

When it left us in 1833, we flattered ourselves with the vain hope that it might not again return. Science was conscious of an enemy, with which she was wholly unprepared to cope. Before sixteen years had elapsed, news of its second approach reached us. Again its course was from east to west; and though far off, from its former history, we could with much assurance, predict its arrival, and almost definitely designate the exact period of its attack, so sure and regular was its course. It reached New Orleans in the winter of 1848–9, gradually extending up the Mississippi and its tributaries, as spring approached; prevailing extensively thoroughout the United States during 1849–50, and in many places to a considerable extent during the summer of '51.

This time it lingered long; was not so fatal or sudden in its attack as in 1832, yet sufficiently so to cause almost universal alarm. The *medical faculty* were but little, if any, better prepared to treat it than formerly. The advantage of sixteen years' reflection, or the sad experience of the past, availed but little. With the exception of a few sporadic cases, we have been exempt from this fatal scourge, now nearly two years.

Again it approaches us from the east, and already many cases have occurred at New Orleans and some at New York; as yet, however, principally confined to recently arrived emigrants from England, where it has prevailed to a considerable extent the past summer. The practice of crowding so many persons (often from six to nine hundred) into our emigrant ships; often destitute of the most ordinary facilities for cleanliness, and having but a meagre supply of even unwholesome food, on which to subsist during a voyage of from six to eight weeks, outraging all laws of health, is so revolting to the better feelings of our nature, that we cannot conceive it possible that those whose business it is, and whose power is adequate to a reformation of so pernicious a practice, will long withhold the remedy.

That this epidemic will again spread through this country, is at least possible. If we may infer its future history from the past, so likely is it, that it behooves medical men everywhere to be prepared to bring to our aid every law of hygiene calculated to stay its approach, modify its attack, or shorten its stay. Although upwards of twenty years have elapsed since we were made familiar with this disorder, yet the very little that is really known concerning it, affords poor encouragement for the future.

The pathologist has labored in vain to discover anything that sheds a single ray of light on its nature, or that enables the physician to institute a rational and successful treatment. Like every other disease the nature of which is imperfectly understood, there are recommended a thousand modes of treatment, each one claiming peculiar potency. Although thus far our management of Cholera has been eminently empirical, certainly with a knowledge of its mode of attack,

(commencing with diarrhœa,) its uniform and rapid tendency to extreme depletion, and many other habitudes belonging to it, we should at least anticipate a more successful practice in future.

Many agents have been tried; some doubtless at times doing good; too often, however, disappointing the anxious hopes of the physician. But of the multitude of agents used, each must make his choice, and in the unsettled and confused state of knowledge of this malady, we can but adopt that course which seems to promise the best success, in view of the surrounding circumstances.

Having already extended this article to too great a length, I shall for the present speak of but two agents—one extensively used in the treatment of Cholera. I allude to opium, usually administered to check diarrhœa. May it not have a more extended effect, by keeping up such a degree of congestion in the cerebral vessels, as to retard the exhaustion of the vital energies of the brain, and thereby prevent or quiet nervous excitation, lessen the severity of the cramps; in short, assist to retard, if not check, the whole train of symptoms arising manifestly from an anæmic state of the blood vessels? The second agent to which I would call attention, is chloroform; which I see is favorably spoken of in the "London Press," as a most potent remedy for allaying cramp in Cholera; and thereby doubtless affording a better chance for the retention and absorption of other remedies, a difficulty that often baffles every attempt at relief.

Keokuk, December, 1853.

The above article contains suggestions of deep moment, not only to medical men, but to the municipal authorities of our cities and populous towns, particularly those on the banks of our principal rivers. From every indication at present, if we are allowed to infer from the past history of that dreadful malady, we may reasonably expect another visit from this destroyer the coming season. Agreeing fully in the opinion expressed by the author of the foregoing, it does behoove us to be prepared as far as possible to mitigate its severity and shorten its stay. Every sanitary measure then should be adopted, and adopted *now*. Measures should be entered upon to prevent the accumulation of noxious materials during the coming winter, and collections of water now existing, should be drained before the warmth of the ensuing spring and summer shall favor their decomposition. Not only so, but all low places, into which water may flow during the winter and spring, should be filled with earth; for it is of doubtful policy, when cess-pools have formed, whether, during the heat of summer, they should be disturbed. It is, however, an unwise policy to wait until it is found they are throwing into the air their poisonous emanations. Not only is it the duty of the "fathers" of each city or town, to attend promptly to this precaution; but it is as necessary for each individual to commence the work of regeneration and purification, about and upon his own premises. Even should we escape a visitation from the Cholera, it will be by no means a useless waste of time, labor or expense.

It is but too true that the efforts of the physician, after the disease has advanced, avail no more now than formerly, and that the proportion of deaths is about the same. There is, however, more known about the pathology of the disease than at first. Enquiry has been on foot, and if our therapeia fail to accomplish more than formerly, we have a better knowledge of the changes that take place in the system during the course of the malady. The experiments of those eminently competent to the task of enquiry, have resulted in the discovery of a marked change in the blood; that there was less fibrine, less water, less albumen, and less saline matters; but more of the red corpuscles than in normal blood. The salts escaped in the serum in solution, the amount found in these profuse discharges from the bowels, bore a near relation to the amount wanting in the blood. Farther experiments showed epithelial portions of the mucous membrane of the bowels mingling in these discharges.

If the opinion of Dr. Stephens be true, that the salts favor, or are the active agents through which the venous is changed to arterial blood, then their escape would prevent this necessary change, and of course the blood would continue its round in the systemic circulation impure. Another fact of importance to be remembered, is that there is but little carbonic acid thrown from the lungs in expiration, and therefore this poisonous element is continued in it to wreak its malignant influences upon the vital forces. Hence the biotic, or brain forces, suffer and continue to fail in proportion as this poison accumulates. In health, a considerable proportion of carbonic acid is thrown off by the lungs, a still larger amount by the skin, the liver and kidneys, and as these depurators are suspended in a great degree, and proportionally to the aggravation of the case, therefore this active poison is retained in the blood, the result of which is an embarrassment of the vital functions, particularly of the nervous centres. The great presiding centre, the brain, especially suffers; and hence a greater or less extinguishment of the biotic forces. In proportion to the activity of the changes of the blood in the lungs, in that proportion is the circulation in the capillaries active; but these changes in this disease are slowly and imperceptibly performed; therefore there is less decarbonization; and yet, because of this poisonous principle, greater activity is demanded. The vessels of the several vital organs, as the substance of the heart, the lungs, the brain and the abdominal viscera, are filled with venous blood, and this also but slightly coagulated.

These changes in the constituents of the blood would point, with almost unerring certainty, to the character of the lesions of function, as well as the therapeutic agents best calculated to remove the morbid condition. However apparently plain the indications, and notwithstanding the boasted assumption of those who believe they have discovered specifics, the mortality is the same as formerly; a fact by no means flattering to the capacities of medical science in the control and management of this "noonday destroyer" of the lives of the human family.

The suggestions with regard to the use of opium are valuable. We have no agent which acts so happily upon the brain in particular conditions, and we are pleased to observe a growing confidence in its ef ficiency, and a less dread of its use. But, as in hemorrhages, it can be used in large and repeated doses, without producing its usual narcotic effects. Dr. Hawthorne, to whom was given a piece of plate in compliment of his success in the treatment of this disease in Europe, gave it in doses of five, ten, and even a higher number of grains at a dose, and repeated it. It would appear that the results were fortunate in his hands. The internal exhibition of chloroform in substance would doubtless prove another valuable agent, but we cannot speak from experience of its use in this disease. But we *can* testify to the valuable service it has rendered in spasmodic affections when given in this manner. Its exhibition by inhalation would be contraindicated, as oxygen would be excluded. For the reasons before alluded to, there is not a sufficient supply taken into the circulation, normally to qualify and oxydize the blood. It would appear necessary to increase the amount of the oxygen of the atmosphere, if possible, rather than to diminish it, and therefore the use of the chloroform in substance into the stomach is doubtless that mode of its exhibition suggested by the writer of the above.

The first question to be settled, is the pathology of Cholera, which will pave the way to the adoption of the most efficient therapeia. A successful prophylactic or hygeine, must be based upon a knowledge of its etiology. These subjects have occupied and are still engaging the attention of the medical mind, and we are pleased that the attention of the profession has been called to the subject by the writer of the above communication. Ed's.

